# Intrauterine infection and postpartum bacteremia due to *Streptococcus gallolyticus subsp gallolyticus*: An emerging concern

**DOI:** 10.1016/j.idcr.2022.e01562

**Published:** 2022-07-02

**Authors:** Sreethish Sasi, Fatma Ben Abid, Godwin Justus Wilson, Ahmed Zaqout, Arun Prabhakaran Nair, P. Chitrambika

**Affiliations:** a Department of Infectious Diseases, Communicable Diseases Centre, Hamad Medical Corporation, Doha, Qatar; b Department of Microbiology, Hamad Medical Corporation, Doha, Qatar; c Department of Anesthesiology, Hamad General Hospital, Hamad Medical Corporation, Doha, Qatar

**Keywords:** SBSEC, *Streptococcus gallolyticus*, Group D streptococci, Postpartum sepsis, Chorioamnionitis

## Abstract

*Streptococcus gallolyticus* is a gram-positive coccus belonging to the family *Streptococcus bovis/Streptococcus equinus complex* (SBSEC). Most cases of SBSEC bacteremia are reported in elderly males with underlying hepatobiliary disease and associated with infective endocarditis (IE) or colonic malignancy. The gastrointestinal tract is the most common portal of entry, followed by the urinary tract and hepatobiliary tree. We present 5 cases of intrapartum bacteremia caused *by S. gallolyticus subsp gallolyticus* reported from the labor unit of our hospital from 2019 to 2021. There was histopathological or microbiological evidence of chorioamnionitis in each case. All the mothers were below the age of 35 years, and none of them had underlying hepatobiliary or colonic disease. All maternal antenatal screenings for *group B streptococci* (GBS) were negative. All the isolates were susceptible to penicillins, ceftriaxone, carbapenems, and vancomycin. Three of them were treated with ceftriaxone and two with aminopenicillins. Duration of treatment varied from 8 days to 14 days. None of the babies were low birth weight or pre-term. All but one baby had clinical sepsis requiring neonatal intensive care unit (NICU) stay, with one having evidence of meningitis and three respiratory distress syndromes (RDS). None of the babies had *S. gallolyticus* bacteremia. All mothers and babies made a complete recovery without any complications. These cases suggest that *S. gallolyticus subsp gallolyticus* can be a rare but emerging cause of intrauterine infection complicated by post-partum bacteremia. There is possibility of colonization of maternal genital tract with *S. gallolyticus* causing neonatal infection.

## Introduction

SBSEC family was formerly known as group D streptococci and included four major species, namely *S. gallolyticus subsp gallolyticus, S. infantarius subsp coli, S. infantarius subsp infantarius,* and *S. gallolyticus subsp pasteurianus.* They are part of the microbiota of the human intestinal tract. SBSEC grows as small nonhemolytic colonies in blood agar. They are catalase-negative and express Lancefield group D [1]. SBSEC are known to cause bacteremia with or without IE in adults. Approximately 5% of bloodstream isolates in hospitalized patients are due to SBSEC [2]. *S. gallolyticus subsp gallolyticus* is more frequently associated with IE than other species. 43–100% of patients with *S. gallolyticus* subsp gallolyticus have IE [3]. Most cases of SBSEC bacteremia are reported in elderly males with underlying hepatobiliary disease and associated with IE or colonic malignancy [4], [5]. The gastrointestinal tract is the most common portal of entry, and other potential sources are the urinary tract and hepatobiliary tree [5]. *S. gallolyticus* as an emerging cause of intrapartum bacteremia and neonatal sepsis, with maternal genitourinary tract being the potential route, is less well studied. Pregnancy is known to cause reticuloendothelial system (RES) dysfunction [6]. Reduced bacterial clearance due to RES dysfunction may enhance their entry into the bloodstream during pregnancy. We present 5 cases of intrapartum bacteremia caused by *S. gallolyticus subsp gallolyticus* reported from the labor unit of our hospital from 2019 to 2021.

## Methods

Data of patients with positive blood cultures of *S. gallolyticus subsp gallolyticus* from 2019 to 2021 was obtained from the microbiology department of Hamad Medical Corporation, Doha, Qatar. From this, women of the reproductive age group (18–45 years) were identified. Electronic medical records of these patients were reviewed to identify parturition-related (intrapartum or postpartum) bacteremia. Five mothers were identified, and their epidemiological and clinical data were collected. Details of the newborns were also obtained from mothers' files, and their data were also collected. Studies involving only data collection are exempted from obtaining informed consent as per institutional review board (IRB) of Hamad Medical Corporation.

In the microbiology laboratory, gram positive cocci from the positive blood cultures were plated and further identified as *Streptococcus gallolyticus subsp gallolyticus* by MALDI-ToF/MS (matrix-assisted laser desorption/ionization time-of-flight mass spectrometry; Bruker Daltonics, Bremen, Germany). An E-test susceptibility (bioMérieux, Durham, NC, USA) was performed and interpreted for penicillin, ceftriaxone, meropenem and vancomycin using Clinical & Laboratory Standards Institute (CLSI) standards.

### Case presentation

Case 1 A 31-year-old female with no significant past medical or surgical history other than *H. Pylori* gastritis with intestinal metaplasia presented with a nuchal cord at 36 weeks. She had two deliveries in the past, one lower segment Cesarian section (LSCS) followed by a vaginal delivery (VBAC). She was planned for vaginal delivery and induced at 37 weeks. Two episodes of fever (38.9 ℃, 39.2 ℃) were noticed during labor, and she was immediately started on intravenous (IV) ceftriaxone 2 g. The initial two sets of blood cultures grew *S. gallolyticus subsp gallolyticus* in both aerobic and anaerobic bottles. Histopathological examination of placenta showed evidence of chorioamnionitis, and tissue culture grew *S. gallolyticus subsp gallolyticus*. The mother did not have any clinical features of meningitis, IE, urinary tract infection (UTI), or gastrointestinal (GI) infection. There was an excellent clinical response. No spikes in fever were noted beyond 48 h of starting ceftriaxone. Repeated blood cultures on day 3 were negative. Her baby was diagnosed to have clinical sepsis and treated with IV amikacin and ampicillin for five days. Both mother and baby made a complete recovery and had no complications at a follow-up after two years.

Case 2 33-year-old primi gravida with no significant past medical or surgical history had spontaneous rupture of membranes at 38 weeks. The fetus was known to be small for gestational age. She had a normal vaginal delivery (NVD) 3 h after rupture of membranes, during which she spiked a fever (39.2 ℃). Blood cultures grew *S. gallolyticus subsp gallolyticus*. Histopathological examination of the placenta showed evidence of chorioamnionitis. She did not have any clinical features of meningitis, IE, urinary tract infection (UTI), or gastrointestinal (GI) infections. A transthoracic echocardiogram (TEE) did not show any vegetations. She was treated successfully with a 7-day course of ceftriaxone. Her baby boy had symptoms suggestive of sepsis and respiratory distress syndrome. Baby’s blood cultures were negative. Both mother and baby recovered well, with no complications at two years.

Case 3 27-year-old primi gravida at 39 weeks of gestation. She was taken for LSCS because of failure of progression of normal labor. Following a spike of fever (39.3 ℃) during labor, her blood and urine cultures grew *S. gallolyticus subsp gallolyticus*. There were no clinical signs of meningitis, or IE and TTE showed no vegetations. She was treated with ten days of ceftriaxone. Her baby boy had tachypnea and respiratory distress and was admitted to the NICU with clinical neonatal sepsis. The baby's blood cultures were negative, but lumbar puncture showed meningitis from cerebrospinal fluid (CSF) cytology and biochemistry. No organism was isolated from the baby's CSF, and he was treated with ten days of IV ampicillin and amikacin.

Case 4 A 33-year-old lady with previous LSCS was taken for repeat LSCS at 41 weeks of gestation because of failure to progress. She had no pre-existing liver or heart conditions. A temperature of 38.8 ℃ was recorded during labor. Blood cultures grew *S. gallolyticus subsp gallolyticus*. A tissue culture of the placenta also grew the same organism. The baby girl had clinical sepsis with a chest X-ray showing pneumonia. No organisms grew in the baby's blood cultures. She was treated with five days of ampicillin and amikacin. Both mother and baby recovered without any complications.

Case 5 A 27-year-old lady with a history of first-trimester miscarriage and antenatal hemorrhage during the current pregnancy was taken for emergency LSCS at 41 weeks. She spiked a fever (38.9 ℃) during labor, and blood cultures grew *S. gallolyticus subsp gallolyticus*. Histopathological examination of fetal membranes showed amnionitis, and no pathologies could be identified in other organ systems. She was treated with 14 days of IV ceftriaxone. Her baby boy did not have sepsis but was treated with empiric IV antibiotics for 48 h until sepsis was ruled out.

A summary of clinical features of the five cases described in this case series is shown in the Table 1.

**Table 1:** summary of clinical features of thefive cases described in this case series*.

	Patient 1	Patient 2	Patient 3	Patient 4	Patient 5
**Age**	31	33	27	33	27
**Year**	2019	2019	2020	2020	2021
**Obstetric History**	G3P2A0L2	Primi	Primi	G2P1A0L1	G2P0A1L0
**Delivery History**	1 LSCS followed by ND	Nil	Nil	Previous 1 LSCS	Previous 1st trimester miscarriage
**Co-morbidities**	Chronic H. Pylori gastritis with intestinal metaplasia	Nil	PCOS	Nil	Nil
**Pre-existing liver disease**	Nil	Nil	Nil	Nil	Nil
**Pre-existing heart conditions**	Nil	Nil	Nil	Nil	Nil
**Antenatal History**	Nuchal cord, 36 weeks	Small for gestational age	Nil	Nil	Antenatal hemorrhage at 34 weeks managed conservatively
**GBS screening**	Negative	Negative	Negative	Negative	Negative
**Gestational Age**	37 weeks	38 weeks	39 weeks	41 weeks	40 weeks
**Type of Delivery**	VBAC	ND	LSCS (failure to progress)	LSCS (failure to progress)	LSCS (Failed trial of Vacuum)
**Intrapartum fever**	Yes	Yes	Yes	Yes	Yes
**Epidural Analgesia**	Yes	Yes	Yes	Yes	Yes
**PROM > 18 h**	No	No	No	No	No
**Onset of fever**	During Labor	During Labor	During Labor	During Labor	During Labor
**Date of first positive blood culture**	Post-partum day 1	Post-partum day 1	Post-partum day 1	Post-partum day 1	Post-partum day 1
**Urine Culture**	Negative	Negative	S. gallolyticus	Negative	Negative
**High Vaginal Swab**	Negative	Negative	Negative	Negative	Negative
**Placental tissue Culture**	S. gallolyticus	Not done	Not done	S. gallolyticus	No growth
**Placenta Histopathology**	Fetal membranes with acute chorioamnionitis	Fetal membranes with acute chorioamnionitis	Amniotic membranes with acute chorioamnionitis Trivascular umbilical cord, focal acute funisitis	Not available	Fetal membranes with mild acute chorioamnionitis
**Repeat blood cultures after 48–72 h**	No growth	No growth	No growth	No growth	No growth
**Susceptibility of S. gallolyticus**	Ceftriaxone-S, Meropenem - S Penicillin – S Vancomycin- S	Ceftriaxone- S Penicillin - S	Ampicillin- S Ceftriaxone- S Penicillin - S	Ceftriaxone- S Penicillin - S	Ceftriaxone- S Penicillin - S
**Signs and Symptoms suggestive of IE**	Nil	Nil	Nil	Nil	Nil
**TTE**	Not done	Not done	No vegetations	No vegetations	No vegetations
**Screening Colonoscopy**	Normal	Normal	Normal	Normal	Normal
**Antibiotic Used**	Oral Cefuroxime (1 day) Oral Amoxicillin-clavulanate (7 days)	Oral Cefuroxime (1 day) IV Ceftriaxone (7 days)	IV Ceftriaxone (10 days)	IV Ampicillin-Sulbactam (6 days), Oral Amoxicillin-Clavulanate (4 days)	IV ceftriaxone (14 days)
**Duration of Antibiotics**	8 days	8 days	10 days	10 days	14 days
**Outcome**	Recovered completely	Recovered completely	Recovered completely	Recovered completely	Recovered completely
**Follow-Up**	No complications at 2 years	No complications at 2 years	No complications at 6 months	No complications at 1 year	No complications at 2 months
**Baby**					
**Sex**	Male	Male	Male	Female	Male
**Birth Weight**	3125 g	2670 g	3620 g	4170 g	3715 g
**Initial Assessment**	Tachycardia, Tachypnea Poor Sucking	Tachycardia, Tachypnea	Tachypnea, Respiratory Distress	Fever, Tachycardia, Tachypnea, Grunting, Chest retractions	Tachycardia
**Blood Cultures**	No growth	No growth	No growth	No growth	No growth
**Neonatal sepsis**	Clinical Sepsis	Respiratory distress syndrome	Respiratory distress syndrome, meningitis	Pneumonia- Right Lower zone infiltrates	No
**Antibiotics**	Amikacin IV – 5 days Ampicillin IV – 5 days	Amikacin IV – 7 days Ampicillin IV – 7 days	Amikacin IV – 10 days Ampicillin IV – 10 days	Amikacin IV – 5 days Ampicillin IV – 5 days	Amikacin IV – 2 days Ampicillin IV – 2 days
**Any other neonatal complications**	No	No	No	No	No
**NICU Stay**	Yes	Yes	Yes	Yes	No
**Follow-Up**	No complications at 2 years	No complications at 2 years	No complications at 1 year	No complications at 1 year	No complications at 2 months

* G – Gravidity, P – Parity, A- Abortions, L- Living Children, LSCS – Lower Segment Cesarian Section, GBS- Group B Streptococcus, VBAC- Vaginal Birth After Cesarian, ND- Normal delivery, IE – Infective Endocarditis, TTE – Trans-thoracic Echocardiogram, IV- Intravenous, NICU- Neonatal Intensive Care Unit, PROM – Premature Rupture of Membrane

## Discussion

*S. gallolyticus* has emerged as an important cause of IE, responsible for 10% of cases in Europe and 6% in the United States [7]. *S. gallolyticus* bloodstream infection is frequently associated with a hepatobiliary origin, and its association with IE and colonic neoplasia is well described [8]. Underlying colonic disease or alterations in the hepatic secretion of bile salts or immunoglobulins may promote the overgrowth of *S. gallolyticus* and its translocation from the intestinal lumen into the portal venous system [5]. The SBSEC fecal carriage rate was 11% among patients with colonic neoplasia [9]. A compromised hepatic reticuloendothelial system may then contribute to the development of *S. gallolyticus* septicemia and subsequent endocarditis [5]. Patients with SBSEC IE usually have relatively large vegetations of more than 10 mm, and it can be highly destructive with valve perforation or septal or valvular ring abscess [10].

The association of *S. gallolyticus* bacteremia with IE and colonic neoplasia in elderly males with an underlying hepatobiliary disease has been well established, and GI or hepatobiliary route is the possible portal of entry [4], [5]. However, *S. gallolyticus subsp gallolyticus* as a pathogen of maternal infection, and its association with newborn infection has not been well studied. In our reported cases, 5 patients had intrapartum infection in which their blood cultures grew *S. gallolyticus subsp gallolyticus*. There was a histopathological or microbiological evidence of chorioamnionitis in each case. All the mothers were below the age of 35 years, and none of them had underlying hepatobiliary or colonic disease. These cases suggest that *S. gallolyticus* subsp gallolyticus can be a rare but emerging cause of intrauterine infection complicated by post-partum bacteremia. Two of the mothers in our case series were primis; one had a first-trimester miscarriage, one had a living child, and another had two. Three mothers had antenatal complications in the form of a nuchal cord, small for gestational age fetus, and third-trimester hemorrhage. Two of them had a normal vaginal delivery, while three had emergency LSCS due to prolonged second stage of labor. All the women spiked fever during the process of delivery. Histopathological examination of the placenta showed chorioamnionitis in four women, and tissue culture grew *S. gallolyticus* in two. Blood cultures of all mothers grew *S. gallolyticus subsp gallolyticus* sensitive to penicillins and cephalosporins. Three of them were treated with ceftriaxone and two with aminopenicillins. Duration of treatment varied from 8 days to 14 days. All mothers made a complete recovery with no complications. None of them had fever spikes beyond 72 h of starting antibiotics, and repeated blood cultures on day 3 were negative. None of the babies were low birth weight or pre-term. All but one had clinical sepsis requiring NICU stay, with one having evidence of meningitis and respiratory distress syndromes (RDS). None of the babies had positive blood cultures; however, they were treated with IV ampicillin and amikacin for five to ten days based on hospital protocol. All babies made a complete recovery.

All SBSEC group bacteria are generally susceptible to penicillins, ceftriaxone, carbapenems, vancomycin, daptomycin, and linezolid, whereas resistance is reported to fluoroquinolones, trimethoprim-sulfamethoxazole, tetracyclines, macrolides, and clindamycin [11]. All our isolates were susceptible to penicillin, ceftriaxone, meropenem and vancomycin. Ceftriaxone (2 g intravenously every 24 h) or a penicillin (12–24 million units intravenously daily, divided every four hours) is preferred to treat *S. gallolyticus* bacteremia. Vancomycin is an acceptable alternative agent for patients with hypersensitivity to beta-lactam agents. Recommended duration of therapy for the treatment of bacteremia is two weeks. Two each of our patients were treated for only eight and ten days, but their outcomes were satisfactory.

Our literature review showed that a case report from China in 2013 first suggested the possibility of intrauterine infection and postpartum bacteremia caused by S. gallolyticus [12]. A microbiological study from China in 2019 evaluated 45 isolates of *S. gallolyticus* from 2012 to 2017. Two out of the 45 (4.44%) patients had bloodstream infection post-delivery with involvement of fetal membranes [13]. Several studies have reported neonatal meningitis and sepsis due to *S. gallolyticus* [14], [15], [16], [17], [18], but none have evaluated the possibility of the maternal genital tract as the possible source of the bacteria. The emergence of S. gallolyticus intrauterine, postpartum, and neonatal infections may be related to its colonization of the maternal genital tract [19]. Antenatal screening for GBS was negative in all our mothers indicating that routine GBS screening may not detect S. gallolyticus. Further studies are needed to know the prevalence of vaginal colonization of S. gallolyticus, risk factors for maternal and neonatal infections, and whether routine antenatal screening is required for the SBSEC group.

Based on the strong epidemiologic with colon cancer, it is recommended that adults with *S. gallolyticus subsp gallolyticus* bacteremia should undergo colonoscopy to evaluate for carcinoma or other colonic lesions. If negative, colonoscopy should be repeated in four to six months. However, the findings of our study suggest that screening colonoscopy may not be mandatory in patients with intrapartum bacteremia due to *S. gallolyticus* unless other indications are present.

## Conclusion

Our observations suggest that *Streptococcus gallolyticus subsp gallolyticus* is emerging as an important cause of intrauterine infection, intrapartum, postpartum, and neonatal bacteremia. Vaginal colonization of the organism should be considered as a possible portal of entry. *S. gallolyticus* may colonize the female lower genital tract, ascend causing secondary intrauterine infection, and have hematogenous seeding due to birth trauma (spontaneous vaginal delivery or surgically induced C-section).

## CRediT authorship contribution statement

Sreethish Sasi**:** Manuscript preparation and literature search. He will act as a study guarantor., Fatma Ben Abid: Patient management, Manuscript review and editing, Mentor, Godwin Justus Wilson: Microbiology data collection, Manuscript writing and editing, Ahmed Zaqout: Patient management, Manuscript review, Arun Prabhakaran Nair: Data collection, Data review, Literature review, Manuscript review and editing, Chitrambika P: Manuscript preparation and literature search.

The authors would like to acknowledge Infectious Diseases Fellowship Program of Hamad Medical Corporation for scientific support.

## Funding sources

The publication of this article was funded by the Qatar National library.

## Ethical approval

The case series was approved by Institutional review board (Medical Research Centre, Hamad Medical Corporation, Doha, Qatar). No informed consent was required for retrospective data review as per policy.

## Consent

No informed consent was required for retrospective data review as per policy.

## Conflict of interest statement

The authors have no conflicts of interest to declare.
